# Reclassification of missense variant pathogenicity using ClinGen recommendations for recalibrated PP3/BP4 in silico predictor score thresholds

**DOI:** 10.1016/j.gimo.2026.104395

**Published:** 2026-03-23

**Authors:** Joseph B. Dubé, Sean Kim, Lynette Lau, Ted Higginbotham, Christian R. Marshall, Rebekah K. Jobling

**Affiliations:** 1 Division of Clinical and Metabolic Genetics, Department of Paediatrics, The Hospital for Sick Children, University of Toronto, Toronto, ON, Canada; 2 Division of Genome Diagnostics, Department of Paediatric Laboratory Medicine, The Hospital for Sick Children, Toronto, ON, Canada; 3 Department of Laboratory Medicine and Pathobiology, University of Toronto, Toronto, ON, Canada

**Keywords:** ACMG/AMP recommendations, ClinGen, PP3/BP4 criteria, Variant effect predictor, Variant interpretation

## Abstract

**Purpose:**

To estimate the effect of ClinGen-calibrated variant effect predictor (VEP) score thresholds on clinically-reported missense variants of uncertain significance (VUS) reclassification using 2015 American College of Medical Genetics/Association for Molecular Pathology variant classification guidelines.

**Methods:**

Missense VUS were reported from clinically indicated genome-wide sequencing or targeted multigene panel testing. Variants were reclassified after integrating select recalibrated VEP pathogenicity scores within the existing 2015 American College of Medical Genetics/Association for Molecular Pathology guidelines. VEPs include VARITY, AlphaMissense, ESM1b, BayesDel, VEST4, REVEL, PolyPhen-2, and SIFT.

**Results:**

Overall median percentage of reclassified missense VUS was 5%. VARITY demonstrated the highest median percent reclassification of missense VUS at approximately 7%. VUS reclassifications from 7 of 8 VEPs demonstrated complete agreement when compared with VUS reclassifications from all other VEPs, except for VARITY which demonstrated a mean percentage agreement of 91%. ESM1b and SIFT demonstrated significantly higher proportions of VUS reclassifications to likely benign compared with likely pathogenic. There was no association between VUS reclassification and autosomal mode of inheritance; however, SIFT and VEST4 demonstrated significantly higher reclassifications of VUS from autosomal dominant genes versus autosomal recessive genes.

**Conclusion:**

Recalibrated VEP pathogenicity score thresholds modestly affect missense VUS reclassification. The included VEPs largely demonstrated equal VUS reclassifications to likely pathogenic or likely benign with near complete agreement in VUS reclassification between the included VEPs.

## Introduction

Within the spectrum of genetic variation reported in typical clinical genetic testing, missense variants—or single-nucleotide variants encoding a change in amino acid residue—are the most frequently reported type of genetic variant.^1^^,^^2^

Missense variants are currently interpreted for clinical significance based on variant classification guidelines established by the American College of Medical Genetics (ACMG) and the Association for Molecular Pathology (AMP) in 2015.^3^ Stringent evidence requirements for estimating pathogenicity or benignity have contributed to a growing abundance of clinically reported missense variants classified as variants of uncertain significance (VUS) for which the indeterminate clinical utility of VUS can complicate diagnosis, management, and genetic counseling.^4^ Clinical variant interpretation currently relies on prior reporting or predictions of variant tolerance based on large population-based sequencing data.^5^ These approaches have well-known limitations including biases in ethnic representation and sensitivity toward variants following an autosomal dominant mode of inheritance.^6^^,^^7^ Furthermore, variant reclassification from VUS to likely pathogenic or pathogenic status can presently take an average of 19 months thus delaying reporting of clinically actionable results.^4^

Computational algorithms that estimate variant pathogenicity, or variant effect predictors (VEPs), are becoming increasingly powerful tools in predicting the clinically relevant impact of individual genomic variants, particularly those variants with limited clinical evidence of pathogenicity. VEP design strategies have advanced alongside recent artificial intelligence-driven strategies such as machine learning and large language models.^8^ VEP performance has been largely assessed by direct concordance between a gold-standard classification method and VEP score.^9^^,^^10^

Under the 2015 ACMG/AMP guidelines, VEP interpretations can contribute the weakest evidence strength level (ie, “Supporting”) under the evidence codes PP4 or BP3 if multiple lines of in silico evidence agreed.^3^ These recommendations were limited given the state of VEPs in 2015 but recognized a role for in silico evidence in clinical variant interpretation. Recently, it was demonstrated that the 2015 ACMG/AMP guidelines conformed to a Bayesian model in which VEP pathogenicity scores could also potentially contribute evidence of pathogenicity at levels greater than “Supporting.”^11^, ^12^, ^13^ Recalibration of top-performing VEPs trained on ClinVar and population-based genomic sequencing databases established discrete VEP-specific score thresholds corresponding to evidentiary strength levels with one VEP demonstrating capability of “Very Strong” evidence of pathogenicity.^11^^,^^14^

At present, the ACMG/AMP is developing a timely reenvisioning of the missense variant classification guidelines, which will incorporate a new points-based system in which VEP interpretation will be incorporated with the possibility of VEP predictors providing stronger evidence than the current “Supporting” level.^12^^,^^15^ We applied the ClinGen recalibrated VEP score thresholds to a clinical data set of reported missense VUS within the current ACMG variant classification framework to estimate the impact of VEP-derived evidence in reclassifying VUS into more clinically actionable classifications.

## Materials and Methods

### Study participants and data

Variants were identified from a heterogenous group of patients with various indications for clinical genetic testing. Sequence variants were identified from either a cohort of patients who received exome or genome sequencing referred to as the genome-wide sequencing (GWS) data set, or multigene panel testing through 1 of 6 next-generation sequencing-based targeted panels (Supplemental Table 1). Variant annotation was based on the GRCh37 genome build. Summary statistics for each genotyping data set is available in Table 1.

**Table 1:** Characteristics for missense variants detected through clinically indicated sequencing

Variant Characteristics	Genetic Testing Data Sets						
	GWS (%)	RD (%)	HL (%)	AI (%)	CT (%)	NS (%)	HSP (%)
***n***	810	542	2472	717	1563	397	720
**Unique genes**	550	41	67	35	79	15	40
**ACMG classifications**							
Benign	1 (0.1)	88 (16)	122 (5)	61 (9)	149 (10)	52 (13)	34 (5)
Likely Benign	0 (0)	43 (8)	141 (6)	52 (7)	92 (6)	14 (4)	39 (5)
VUS	604 (75)	335 (62)	2130 (86)	590 (82)	1245 (80)	228 (57)	610 (85)
Likely pathogenic	122 (15)	66 (12)	57 (2)	11 (2)	48 (3)	28 (7)	19 (3)
Pathogenic	83 (10)	10 (2)	22 (1)	3 (0)	29 (2)	75 (19)	18 (3)
**In silico Evidence Use**							
PP3	303 (37)	165 (30)	849 (34)	136 (19)	438 (28)	174 (44)	208 (29)
BP4	173 (21)	122 (23)	445 (18)	235 (33)	310 (20)	29 (7)	185 (26)

*AI*, autoinflammatory gene panel; *CT*, connective tissue disorders gene panel; *GWS*, genome-wide sequencing; *HL*, hearing loss gene panel; *HSP*, hereditary spastic paraplegia gene panel; *n*, number of unique missense variants; *NS*, Noonan syndrome gene panel; *RD*, renal disease gene panel.

### Missense variant VEP score annotation and reclassification

Missense variants were identified by the expected effect on the encoded change in amino acid residue based on the Matched Annotation from NCBI and EMBL-EBI Select transcripts.^16^ Baseline variant interpretations were manually curated by the Genome Diagnostics Laboratory, which provided clinical variant classifications according to the ACMG/AMP guidelines.^3^

VEPs included in this study were previously reanalyzed by ClinGen with recalibrated thresholds for VEP scores corresponding to ACMG 2015 evidentiary strength levels (eg, Pathogenic—very strong, strong, moderate, and supporting).^11^^,^^14^ OpenCRAVAT (https://www.opencravat.org) is a publicly available online annotation tool that assigned VEP scores to each variant based on the hg19 genome build.^17^ Selected VEPs included SIFT and PolyPhen 2, which are long-standing and widely utilized VEPs.^18^^,^^19^ Based on the ACMG 2022 meeting presentation on the upcoming revised variant classification guidelines, we included BayesDel, VEST4, and REVEL.^20^, ^21^, ^22^ More recent state-of-the-art VEPs, including ESM1b, AlphaMissense, and VARITY, have been recalibrated and were included in this study.^14^^,^^23^, ^24^, ^25^ Precomputed VEP scores for the MutPred2 algorithm were not publicly available while preparing this manuscript.

Missense variant reclassification was performed only on variants where initial classification included BP3 or PP4 evidence. These select variants were reclassified by comparing a given VEP score with the corresponding ClinGen recalibrated score thresholds to determine whether a variant may be annotated with a BP3 or PP4 evidence strength level greater than “supporting” (eg, PP3_Strong or BP4_Very Strong). Variant classifications were then recalculated according to the ACMG 2015 guidelines and incorporating the same non-VEP evidence sources used by the laboratory to determine the initial variant classification. Instances where BP3 or PP4 were applied to the initial variant classification by the laboratory were replaced with the revised alternate BP3 or PP4 evidentiary strengths where appropriate to avoid inflating BP3- or PP4-related evidence. Variant reclassification percentages represent the proportion of variants that demonstrated a classification change after the application of ClinGen recommendations.

### Statistical analysis

RStudio Version 2025.05.0 was used for all statistical analyses.^26^ Variant reclassification percentages across the different clinical data sets were calculated by comparing the change in classification pre- and postrevised PP3 and BP4 evidence. Odds ratios (ORs) were calculated using Fisher’s Exact Test where appropriate. Agreement between VEPs was also assessed by comparing whether instances of variant reclassification based on one VEP score was similarly demonstrated across the other VEPs included in this study.

## Results

### Composition of sequencing data sets and application of PP3 and BP4 evidence

Table 1 details the number of unique missense variants and the number of unique genes across clinical test indications. The GWS data set included the largest number of unique genes, whereas the targeted gene panel data sets had expectedly smaller numbers of unique genes. The frequencies of missense variant classifications following the 2015 ACMG/AMP guidelines consistently demonstrated a majority of VUS across all data sets where the GWS data set had a significantly lower likelihood of reporting VUS compared with all gene panel data sets (OR 0.73, 95% CI 0.61-0.86). The GWS data set featured a larger percentage of likely pathogenic (LP) and pathogenic variants and the smallest number of likely benign (LB) and benign variants compared with the targeted gene panel data sets. Application of either PP3 or BP4 rules was frequent among all data sets where the GWS data set had the highest application of PP3/BP4 evidence in 58% of variants. Usage of PP3 or BP4 within the targeted gene panel data sets was relatively lower and ranged from 48% to 53%.

### Impact of applying recalibrated VEP score thresholds on missense VUS reclassification

**Figure 1:**
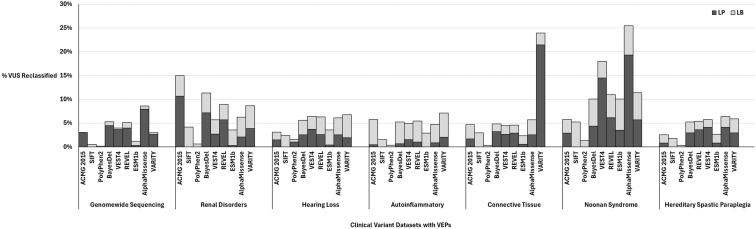
**Comparison of VEP-specific VUS reclassification percentages across clinical sequencing data sets.** Percent missense VUS reclassification is organized by VEP on the *x*-axis with vertical labels. ACMG 2015 refers to application of the ACMG/AMP 2015 variant classification criteria. Bars are segmented based on the proportion of VUS reclassified as LP (dark) or LB (light) segments. The *x*-axis is further grouped based on the clinical sequencing data set with horizontal labels. LB, likely benign; LP, likely pathogenic; VEP, variant effect predictor; VUS, variant of uncertain significance.

Figure 1 demonstrates the percentages of missense VUS that were reclassified to either LP or LB classifications after the integration of the recalibrated VEP score thresholds within the 2015 ACMG/AMP guidelines. Overall, the median percentage of VUS reclassified across all data sets and VEPs was 5% with the large majority of VUS classifications remaining unchanged.

Comparing sequencing data sets, the GWS dataset had the lowest median percentage of reclassified VUS of approximately 3%, whereas the Noonan Syndrome targeted gene panel had the highest median percentage of 10%. The proportion of missense VUS reclassifications to LP versus LB was greatest within the GWS data set in which LP reclassifications comprised approximately 84% of missense VUS reclassifications. This contrasted with some of the targeted gene panels, including the Renal Disorders panel, in which approximately 51% of missense VUS reclassifications were LP, or the Autoinflammatory Disorders panel which demonstrated the lowest percentage of VUS reclassification to LP among the data sets with approximately 17% of missense VUS reclassified to LP.

We assessed VEP performance across each data set and calculated a median percentage of VUS reclassified. VARITY demonstrated the highest median percent reclassification of missense VUS at approximately 7%, whereas REVEL, BayesDel, VEST4, and AlphaMissense median percentage reclassification ranged from 5% to 6%, although this difference was not statistically significant. Interestingly, ESM1b had an average of 4%, which was also not significantly different compared with VARITY. SIFT and Polyphen-2 demonstrated the lowest average percentages for missense VUS reclassification at 3% and 1%, respectively, which was significantly lower compared with VARITY. We estimated the contribution of the 2015 ACMG/AMP recommended use of PP3 /BP4 evidence by removing instances of PP3/BP4 evidence in the baseline laboratory-derived variant classifications, which demonstrated an approximate 6% average missense VUS reclassification.

Although the vast majority of missense VUS were reclassified into either LP or LB classifications, 6 missense VUS were reclassified to Benign classifications (Table 2). All 6 variants were identified on the Hearing Loss panel involving 6 unique genes. Five of the 6 VUS were annotated with VARITY scores that reached a recalibrated threshold of BP4_Strong, whereas 1 VUS was annotated with a REVEL score that also corresponded with a recalibrated score threshold of BP4_Strong. No instances of VUS being reclassified to Pathogenic were identified in this study.

**Table 2:** Missense variants of uncertain significance reclassified to Benign using recalibrated VEP thresholds

Panel	Gene	HGNC	Location	MANE Select Transcript	VEP	VEP Score	Evidence Strength	Reclassification
HL	*ADGRV1*	17416	NC_000005.9: g.89938764A>G	NM_032119.4:c.2459A>G	REVEL	0.014	BP4_Strong	VUSàB
HL	*USH2A*	12601	NC_000001.10:g.216011445C>T	NM_206933.4:c.9259G>A	VARITY	0.032904	BP4_Strong	VUSàB
HL	*WFS1*	12762	NC_000004.11:g.6279355C>T	NM_006005.3:c.173C>T	VARITY	0.021316	BP4_Strong	VUSàB
HL	*GSDME*	2810	NC_000007.13:g.24784260C>T	NM_001127453.2:c.325G>A	VARITY	0.020728	BP4_Strong	VUSàB
HL	*CHD7*	20626	NC_000008.10:g.61765978A>G	NM_017780.4:c.6694A>G	VARITY	0.012199	BP4_Strong	VUSàB
HL	*MYH14*	23212	NC_000019.9:g.50783609C>T	NM_001145809.2:c.4259C>T	VARITY	0.019462	BP4_Strong	VUSàB

*B*, benign; *Chr*, chromosome; *HGNC*, HUGO Gene Nomenclature Committee gene ID; *HL*, hearing loss gene panel; *Location*, chromosome and genomic position per GRCh37 genome build; *VEP*, variant effect predictor; *VUS*, variant of uncertain significance.

Mode of inheritance (MOI) is a major factor modifying variant pathogenicity but is not typically considered in VEP algorithms. We examined missense VUS reclassifications based on expected autosomal dominant (AD) or recessive (AR) MOI. There was an insufficient number of missense VUS of X-linked MOI for statistical analysis. SIFT and VEST4 demonstrated significantly higher percentages of reclassified AD VUS of approximately 3.2% and 8.3%, respectively. Among the remaining VEPs, there was no significant difference between AD or AR VUS reclassifications. Only the Hereditary Spastic Paraplegia gene panel dataset demonstrated a significant 6-fold higher mean percentage of reclassified AD VUS compared with AR VUS, whereas there was no significant difference in mean AD or AR VUS reclassifications within the other data sets.

### Assessing agreement between VEP reclassifications in the GWS data set

Table 3 demonstrates the total number of missense VUS reclassifications in which any 2 VEPs agreed. Numbers in parentheses demonstrate the percentage of concordant missense VUS reclassifications out of the total number of missense VUS reclassified between any 2 VEPs. Overall, 64% of all 196 inter-VEP comparisons demonstrated 100% concordance.

**Table 3:** Concordance between VEPs on missense VUS reclassifications

VEP	VEP Comparator	Data Sets						
		GWS (%)	RD (%)	HL (%)	AI (%)	CT (%)	NS (%)	HSP (%)
**VARITY**								
	**AlphaMissense**	27 (96.43)	29 (85.29)	107 (90.68)	43 (82.69)	78 (97.5)	34 (97.14)	31 (91.18)
	**ESM1b**	11 (84.62)	22 (81.48)	75 (88.24)	31 (79.49)	43 (95.56)	25 (96.15)	20 (86.96)
	**REVEL**	19 (95)	45 (90)	106 (92.17)	49 (83.05)	65 (97.01)	26 (96.3)	27 (93.1)
	**VEST4**	15 (88.24)	32 (88.89)	92 (91.09)	40 (81.63)	64 (96.97)	21 (95.45)	22 (91.67)
	**BayesDel**	17 (94.44)	49 (90.74)	97 (90.65)	9 (100)	63 (96.92)	24 (96)	24 (92.31)
	**SIFT**	4 (80)	26 (86.67)	58 (86.57)	25 (75.76)	47 (95.92)	15 (93.75)	13 (81.25)
	**Polyphen-2**	0 (0)	6 (75)	15 (83.33)	10 (71.43)	16 (94.12)	6 (85.71)	2 (40)
**AlphaMissense**								
	**ESM1b**	18 (94.74)	29 (100)	80 (100)	36 (100)	55 (100)	29 (100)	23 (100)
	**REVEL**	32 (100)	27 (96.43)	99 (98.02)	48 (97.96)	71 (100)	36 (100)	29 (100)
	**VEST4**	21 (95.45)	29 (100)	106 (100)	45 (100)	80 (100)	54 (100)	27 (100)
	**BayesDel**	37 (100)	33 (100)	101 (100)	5 (100)	71 (100)	30 (100)	29 (100)
	**SIFT**	6 (100)	38 (100)	68 (100)	34 (100)	58 (100)	18 (100)	18 (100)
	**Polyphen-2**	1 (100)	7 (100)	16 (100)	14 (100)	21 (100)	8 (100)	5 (100)
**ESM1b**								
	**REVEL**	15 (100)	23 (95.83)	72 (97.3)	35 (97.22)	47 (100)	22 (100)	20 (100)
	**VEST4**	12 (100)	23 (100)	79 (100)	34 (100)	50 (100)	21 (100)	16 (100)
	**BayesDel**	16 (100)	27 (100)	74 (100)	2 (100)	46 (100)	25 (100)	16 (100)
	**SIFT**	7 (100)	27 (100)	61 (98.39)	30 (100)	39 (100)	18 (100)	15 (100)
	**Polyphen-2**	1 (100)	8 (100)	16 (100)	11 (100)	17 (100)	7 (100)	6 (100)
**REVEL**								
	**VEST4**	25 (100)	39 (97.5)	101 (98.06)	42 (97.67)	75 (100)	28 (100)	30 (100)
	**BayesDel**	42 (100)	59 (98.33)	128 (98.46)	6 (100)	92 (100)	27 (100)	38 (100)
	**SIFT**	4 (100)	22 (95.65)	47 (94)	31 (96.88)	41 (100)	15 (100)	14 (100)
	**Polyphen-2**	1 (100)	6 (85.71)	17 (100)	15 (100)	19 (100)	8 (100)	5 (100)
**VEST4**								
	**BayesDel**	19 (100)	43 (100)	107 (100)	8 (100)	80 (100)	24 (100)	30 (100)
	**SIFT**	3 (75)	24 (100)	55 (98.21)	31 (100)	45 (100)	12 (100)	12 (100)
	**Polyphen-2**	1 (100)	5 (100)	17 (100)	11 (100)	21 (100)	8 (100)	5 (100)
**BayesDel**								
	**SIFT**	6 (100)	30 (100)	51 (98.08)	3 (100)	41 (100)	16 (100)	15 (100)
	**Polyphen2**	1 (100)	7 (100)	17 (100)	1 (100)	21 (100)	8 (100)	4 (100)
**SIFT**								
	**Polyphen2**	1 (100)	7 (100)	16 (100)	13 (100)	19 (100)	7 (100)	5 (100)

*AI*, autoinflammatory panel; *CT*, connective tissue disorders panel; *GWS*, genome-wide sequencing; *HL*, hearing loss panel; *HSP*, hereditary spastic paraplegia panel; *NS*, Noonan syndrome panel; *RD*, renal disorders panel.

In terms of individual VEP performance, nearly all VEPs demonstrated 100% median percentage concordance with exception of VARITY, which demonstrated a median percentage concordance of 91%.

Nearly all data sets demonstrated 100% median percentage concordance for all inter-VEP comparisons with the exception of the Hearing Loss gene panel which demonstrated median percentage concordance of 98%.

## Discussion

In this study, we incorporated recently recalibrated VEP score thresholds within the current 2015 ACMG/AMP missense variant classification guidelines and estimated the overall impact of recommended select VEP scores on missense VUS reclassification to be approximately 5% with noted variability between datasets and individual VEPs. We also found 64% of all inter-VEP comparisons for missense VUS reclassifications showed total concordance. Interestingly, VARITY was the only VEP to demonstrate median percentage concordance below 100% considering all comparisons with non-VARITY VEPs across all datasets.

Our study adopted a unique approach to assessing the impact of VEP-derived missense VUS reclassification by reanalyzing the ACMG/AMP 2015 evidence manually curated by our laboratory and modifying BP4/PP3 evidence according to the recalibrated VEP score thresholds utilizing multiple of clinically derived sequencing data sets. VEP performance has largely involved analyzing receiver-operator characteristic curves and various large-scale genetic variant data sets typically involving ClinVar data as a gold standard and not in terms of variant reclassification.^9^^,^^10^^,^^23^^,^^27^, ^28^, ^29^ Previous studies have examined the broader impact of a Bayesian-derived point-based system compared with the ACMG/AMP 2015 guidelines in which Wilcox et al^30^ observed that PP3 evidence contributed to reclassifying approximately 14% of select missense VUS and LP variants for which application of PP3 or BP4 evidence within the point-based framework contributed to the final classification of approximately 16% of variants examined.^12^^,^^30^ Eldomery et al^31^ applied a version of the Bayesian point-based classification system to hereditary cancer predisposition genes for which the percentage of VUS classifications decreased by 21% compared with the 2015 ACMG/AMP guidelines. Elsewhere, Stenton et al^32^ assessed the evidence yield for recalibrated BP4/PP3 recommendations by reanalyzing missense variants from genome sequences of 300 probands and determined that a likely limited proportion of variants per proband would demonstrate VEP scores surpassing a “Supporting” level of evidence.

Our methodological approach examined the impact on variant reclassification by integrating the ClinGen recalibrated VEP score thresholds within the current ACMG/AMP 2015 guidelines. By performing this analysis within diverse sequencing data sets derived from medically indicated genetic testing, we believe that our approach provides an accurate estimation of how the new recommendations for the application of BP4/PP3 evidence impact clinical missense VUS reclassification. Assuming 5% of missense VUS in clinically determined VUS can be reclassified to a clinically actionable classification, our results suggest that new recommendations for VEP score utilization will likely contribute modestly to reducing VUS classifications as previously hypothesized.^32^

Only 2 VEPs included in our study, SIFT and VEST4, demonstrated significantly higher VUS reclassifications for variants from AD genes versus AR genes suggesting no obvious bias in VUS reclassification based on MOI. Of the data sets studied, only the Hereditary Spastic Paraplegia gene panel data set demonstrated a significantly higher proportion of VUS reclassifications from AD-related genes versus AR-related genes. SIFT and VEST4 are 2 fundamentally distinct VEPs with the former based on homologous sequence alignment comparisons and the latter representing a supervised machine learning-based model incorporating genomic and functional data.^18^^,^^21^ Although our results did not support consistent MOI-associated VUS reclassification, MOI has previously demonstrated to improve variant interpretation and may represent a potentially under-utilized parameter for future variant interpretation algorithms.^33^

Agreement between VEPs for missense VUS reclassification was generally high with median percentage concordance of 100% for all VEPs except for VARITY. VARITY is a more recently developed meta-VEP that has been benchmarked as a top-performing VEP.^25^ It was beyond the scope of this study to investigate factors underlying why VARITY demonstrated variable discordance with all other VEPs across each data set; however, our findings may suggest unique algorithmic features affecting scores for certain variants, which is discordant with multiple independent VEPs. Unique intragenic architectural features, such as intrinsically disordered regions, have been proposed as one factor that is variably present throughout the genome and likely affects VEP performance.^34^ Accordingly, further study and replication is warranted. As Figure 1 demonstrated, missense VUS reclassification percentages were typically comparable between VEPs within a given data set with similar proportions of VUS being reclassified to LP or LB. This suggests that, overall, the selected VEPs in our study tend to agree on missense VUS reclassifications, which may be reassuring at a time when numerous VEPs are freely available and there is no clear consensus on which VEPs should be prioritized for clinical variant reclassification. Our study results do suggest, however, that previous-generation VEPs such as SIFT and PolyPhen2 should not be prioritized ahead of newer recalibrated top-performing VEPs.

Our analysis of VEP performance between data sets suggested a consistent difference between the GWS data set and targeted gene panels particularly the Noonan Syndrome panel. The lower VUS reclassification percentage observed for the GWS data set may represent a more accurate estimation of the impact of the revised VEP threshold scores on VUS reclassification given that the GWS data set comprised genome-wide VUS from a heterogenous patient population. We showed that VEPs demonstrated a high degree of agreement when VUS were reclassified, which was also seen in the Noonan Syndrome (OMIM 163950) gene panel VUS reclassifications. Six of 8 VEPs consistently reclassified Noonan Syndrome variants at approximately twice the overall variant reclassification percentage where an average of approximately 54% of reclassified VUS were located within the neurofibromin gene (*NF1*). *NF1* variants may have a propensity for predicted pathogenicity because of a combination of intrinsic gene-specific characteristics, such as loci-specific estimations of variation tolerance, amino acid composition, and predicted impact on regional protein features. These findings suggest that reclassified *NF1* VUS underly the disproportionate VUS reclassification in the Noonan Syndrome panel. Frequent *NF1* VUS reclassifications may also be associated with strong clinical suspicion and genotype-phenotype correlation, or intrinsic locus-specific features as described above that may affect predicted pathogenicity. Discordant locus-specific VUS reclassification percentages may also highlight a risk for VEP overestimation of VUS pathogenicity. There is currently a lack of robust benchmarking tools to evaluate VEP performance in VUS reclassification. However; this issue may be addressed in part by further recalibrating VEP scores based on a more targeted locus-specific approach.^35^, ^36^, ^37^, ^38^ Additionally, functional assays and multiplexed assays of variant effect represent a promising opportunity to further develop VEP algorithms or separately refine functional evidence within the ACMG/AMP variant classification criteria.^39^

We recognize limitations in our study design. First, we utilized the ACMG/AMP 2015 framework for variant classification because the proposed point-based classification system was not available at the time of manuscript preparation. It will be important to replicate our study utilizing the upcoming points-based framework to better understand the contribution of VEPs in variant classification. Second, our study likely featured a proportion of variants previously used to train VEPs featured in this study. Although this raises the issue of data circularity and a risk for skewing VEP scores, VEPs typically utilize statistical approaches such as out-of-bag sampling to address data circularity.^20^ It is also unavoidable to uncover clinical variants that were used in VEP training. Both current and future applications of PP3 and BP4 evidence are guided by consistent VEP application in which the upcoming revised ACMG/AMP classification criteria will likely emphasize selecting 1 VEP within a point-based system as opposed to interchanging VEPs.^15^

Lastly, a growing number of in silico predictors examine variants in noncoding regions, such as splice sites or promoter regions.^40^^,^^41^ Going forward, clinical variant classification and prioritization will likely incorporate in silico algorithms capable of interpreting the broader spectrum of genomic variation.

### Conclusions

Recalibrated VEP score thresholds have an overall modest effect on clinical missense VUS reclassification; however, this may vary based on the specific VEP or clinical indication for genetic testing. Selected VEPs included in this study demonstrated high concordance regarding missense VUS reclassifications. VEPs will continue to play important role in clinical variant interpretation while the optimal integration of VEPs within clinical variant classification protocols remains to be determined.

## Conflict of Interest

The authors declare no conflicts of interest.
