# Supplementary material for: Reclassification of missense variant pathogenicity using ClinGen recommendations for recalibrated PP3/BP4 in silico predictor score thresholds
Source: Genet Med Open. 2026 Mar 23;4:104395. doi: 10.1016/j.gimo.2026.104395 (PMC13125162; doi:10.1016/j.gimo.2026.104395)
Supplement: Supplementary Table 1 [file mmc1.docx]

**Supplemental Table 1. Genetic loci included within targeted gene panel datasets.**

| **Gene** | **Panel** | **Panel2** | **HGNC** | **Gene** | **Panel** | **Panel2** | **HGNC** |
| --- | --- | --- | --- | --- | --- | --- | --- |
| AP3B1 | AI |  | 566 | B4GALT7 | CT |  | 930 |
| ARPC1B | AI |  | 704 | BMP1 | CT |  | 1067 |
| BLOC1S6 | AI |  | 8549 | CA2 | CT |  | 1373 |
| CARD14 | AI |  | 16446 | CBS | CT |  | 1550 |
| CD27 | AI |  | 11922 | CHST14 | CT |  | 24464 |
| ADA2 | AI |  | 1839 | CLCN7 | CT |  | 2025 |
| COPA | AI |  | 2230 | COL11A1 | CT | HL | 2186 |
| ELANE | AI |  | 3309 | COL1A2 | CT |  | 2198 |
| IL1RN | AI |  | 6000 | COL2A1 | CT | HL | 2200 |
| IL36RN | AI |  | 15561 | COL3A1 | CT |  | 2201 |
| ITK | AI |  | 6171 | COL5A1 | CT |  | 2209 |
| LACC1 | AI |  | 26789 | COL5A2 | CT |  | 2210 |
| LPIN2 | AI |  | 14450 | COL9A1 | CT | HL | 2217 |
| LYST | AI |  | 1968 | COL9A2 | CT | HL | 2218 |
| MEFV | AI |  | 6998 | COMP | CT |  | 2227 |
| MVK | AI |  | 7530 | CRTAP | CT |  | 2379 |
| NLRP12 | AI |  | 22938 | DDR2 | CT |  | 2731 |
| NOD2 | AI |  | 5331 | DSE | CT |  | 21144 |
| PLCG2 | AI |  | 9066 | DYM | CT |  | 21317 |
| PRF1 | AI |  | 9360 | EBP | CT |  | 3133 |
| PSMB8 | AI |  | 9545 | EIF2AK3 | CT |  | 3255 |
| PSTPIP1 | AI |  | 9580 | FBN2 | CT |  | 3604 |
| RAB27A | AI |  | 9766 | FGFR3 | CT |  | 3690 |
| RBCK1 | AI |  | 15864 | FKBP10 | CT |  | 18169 |
| RIPK1 | AI |  | 10019 | FKBP14 | CT |  | 18625 |
| SH2D1A | AI |  | 10820 | FLNB | CT |  | 3755 |
| SH3BP2 | AI |  | 10825 | HSPG2 | CT |  | 5273 |
| SLC7A7 | AI |  | 11065 | IFITM5 | CT |  | 16644 |
| STX11 | AI |  | 11429 | IFT122 | CT |  | 13556 |
| STXBP2 | AI |  | 11445 | IFT43 | CT |  | 29669 |
| STING1 | AI |  | 27962 | IFT80 | CT |  | 29262 |
| TNFAIP3 | AI |  | 11896 | LBR | CT |  | 6518 |
| TNFRSF1A | AI |  | 11916 | LIFR | CT |  | 6597 |
| UNC13D | AI |  | 23147 | LRP5 | CT |  | 6697 |
| XIAP | AI |  | 592 | MATN3 | CT |  | 6909 |
| ACTA2 | CT |  | 130 | MBTPS2 | CT |  | 15455 |
| ADAMTS2 | CT |  | 218 | NEK1 | CT |  | 7744 |
| ALPL | CT |  | 438 | NKX3-2 | CT |  | 951 |
| ARSL | CT |  | 719 | NSDHL | CT |  | 13398 |
| ATP7A | CT |  | 869 | OSTM1 | CT |  | 21652 |
| **Gene** | **Panel** | **Panel2** | **HGNC** | **Gene** | **Panel** | **Panel2** | **HGNC** |
| P3H1 | CT |  | 19316 | COL4A3 | HL | RD | 2204 |
| PEX7 | CT |  | 8860 | COL4A4 | HL | RD | 2206 |
| PLEKHM1 | CT |  | 29017 | COL4A5 | HL | RD | 2207 |
| PLOD1 | CT |  | 9081 | DIAPH1 | HL |  | 2876 |
| PLOD2 | CT |  | 9082 | EDN3 | HL |  | 3178 |
| PLS3 | CT |  | 9091 | EDNRB | HL |  | 3180 |
| PPIB | CT |  | 9255 | ESPN | HL |  | 13281 |
| PRDM5 | CT |  | 9349 | EYA1 | HL |  | 3519 |
| PTH1R | CT |  | 9608 | EYA4 | HL |  | 3522 |
| SERPINF1 | CT |  | 8824 | GJB2 | HL |  | 4284 |
| SERPINH1 | CT |  | 1546 | GJB6 | HL |  | 4288 |
| SLC26A2 | CT |  | 10994 | GPSM2 | HL |  | 29501 |
| SLC35D1 | CT |  | 20800 | GRHL2 | HL |  | 2799 |
| SLC39A13 | CT |  | 20859 | GRXCR1 | HL |  | 31673 |
| SMAD3 | CT |  | 6769 | GSDME | HL |  | 2810 |
| SOX9 | CT |  | 11204 | HGF | HL |  | 4893 |
| SP7 | CT |  | 17321 | ILDR1 | HL |  | 28741 |
| SPARC | CT |  | 11219 | KCNE1 | HL |  | 6240 |
| TCIRG1 | CT |  | 11647 | KCNQ1 | HL |  | 6294 |
| TGFB2 | CT |  | 11768 | KCNQ4 | HL |  | 6298 |
| TGFBR1 | CT |  | 11772 | LHFPL5 | HL |  | 21253 |
| TGFBR2 | CT |  | 11773 | LOXHD1 | HL |  | 26521 |
| TMEM38B | CT |  | 25535 | LRTOMT | HL |  | 25033 |
| TNFRSF11A | CT |  | 11908 | MARVELD2 | HL |  | 26401 |
| TNFRSF11B | CT |  | 11909 | MITF | HL |  | 7105 |
| TNFSF11 | CT |  | 11926 | MYH14 | HL |  | 23212 |
| TRAPPC2 | CT |  | 23068 | MYH9 | HL | RD | 7579 |
| TRIP11 | CT |  | 12305 | MYO15A | HL |  | 7594 |
| TRPV4 | CT |  | 18083 | MYO3A | HL |  | 7601 |
| TTC21B | CT |  | 25660 | MYO7A | HL |  | 7606 |
| WDR19 | CT |  | 18340 | OTOA | HL |  | 16378 |
| WDR35 | CT |  | 29250 | OTOF | HL |  | 8515 |
| WNT1 | CT |  | 12774 | PAX3 | HL |  | 8617 |
| XYLT2 | CT |  | 15517 | PCDH15 | HL |  | 14674 |
| ACTG1 | HL |  | 144 | PDZD7 | HL |  | 26257 |
| ADGRV1 | HL |  | 17416 | PJVK | HL |  | 29502 |
| CDH23 | HL |  | 13733 | PRPS1 | HL |  | 9462 |
| CHD7 | HL |  | 20626 | RDX | HL |  | 9944 |
| CIB2 | HL |  | 24579 | SERPINB6 | HL |  | 8950 |
| CLDN14 | HL |  | 2035 | SIX1 | HL |  | 10887 |
| CLRN1 | HL |  | 12605 | SLC17A8 | HL |  | 20151 |
| COCH | HL |  | 2180 | SMPX | HL |  | 11122 |
| **Gene** | **Panel** | **Panel2** | **HGNC** | **Gene** | **Panel** | **Panel2** | **HGNC** |
| SOX10 | HL |  | 11190 | SACS | HSP |  | 10519 |
| STRC | HL |  | 16035 | SETX | HSP |  | 445 |
| TCOF1 | HL |  | 11654 | SLC16A2 | HSP |  | 10923 |
| TECTA | HL |  | 11720 | SPAST | HSP |  | 11233 |
| TMC1 | HL |  | 16513 | SPG11 | HSP |  | 11226 |
| TMPRSS3 | HL |  | 11877 | SPG20 | HSP |  | 18514 |
| TPRN | HL |  | 26894 | SPG21 | HSP |  | 20373 |
| TRIOBP | HL |  | 17009 | SPG7 | HSP |  | 11237 |
| USH1C | HL |  | 12597 | TECPR2 | HSP |  | 19957 |
| USH1G | HL |  | 16356 | UCHL1 | HSP |  | 12513 |
| USH2A | HL |  | 12601 | VPS13D | HSP |  | 23595 |
| WFS1 | HL |  | 12762 | BRAF | NS |  | 1097 |
| WHRN | HL |  | 16361 | CBL | NS |  | 1541 |
| ALDH18A1 | HSP |  | 9722 | HRAS | NS |  | 5173 |
| ALS2 | HSP |  | 443 | KRAS | NS |  | 6407 |
| AP4B1 | HSP |  | 572 | MAP2K1 | NS |  | 6840 |
| AP4E1 | HSP |  | 573 | MAP2K2 | NS |  | 6842 |
| AP4M1 | HSP |  | 574 | NF1 | NS |  | 7765 |
| ATL1 | HSP |  | 11231 | NRAS | NS |  | 7989 |
| BSCL2 | HSP |  | 15832 | PTPN11 | NS |  | 9644 |
| MTRFR | HSP |  | 26784 | RAF1 | NS |  | 9829 |
| CYP2U1 | HSP |  | 20582 | RIT1 | NS |  | 10023 |
| CYP7B1 | HSP |  | 2652 | SHOC2 | NS |  | 15454 |
| DDHD1 | HSP |  | 19714 | SOS1 | NS |  | 11187 |
| DDHD2 | HSP |  | 29106 | SOS2 | NS |  | 11188 |
| ERLIN2 | HSP |  | 1356 | SPRED1 | NS |  | 20249 |
| FA2H | HSP |  | 21197 | ACTN4 | RD |  | 166 |
| FARS2 | HSP |  | 21062 | COQ8B | RD |  | 19041 |
| GBA2 | HSP |  | 18986 | C3 | RD |  | 1318 |
| HACE1 | HSP |  | 21033 | CD2AP | RD |  | 14258 |
| HSPD1 | HSP |  | 5261 | CD46 | RD |  | 6953 |
| WASHC5 | HSP |  | 28984 | CEP290 | RD |  | 29021 |
| KIF1A | HSP |  | 888 | CFB | RD |  | 1037 |
| KIF1C | HSP |  | 6317 | CFHR2 | RD |  | 4890 |
| KIF5A | HSP |  | 6323 | CFHR3 | RD |  | 16980 |
| L1CAM | HSP |  | 6470 | CFHR4 | RD |  | 16979 |
| NT5C2 | HSP |  | 8022 | CFHR5 | RD |  | 24668 |
| PLP1 | HSP |  | 9086 | CFI | RD |  | 5394 |
| PNPLA6 | HSP |  | 16268 | GLIS2 | RD |  | 29450 |
| POLG | HSP |  | 9179 | INF2 | RD |  | 23791 |
| REEP1 | HSP |  | 25786 | INVS | RD |  | 17870 |
| RTN2 | HSP |  | 10468 | LAMB2 | RD |  | 6487 |
| **Gene** | **Panel** | **Panel2** | **HGNC** |  |  |  |  |
| LMX1B | RD |  | 6654 |  |  |  |  |
| NEK8 | RD |  | 13387 |  |  |  |  |
| NPHP1 | RD |  | 7905 |  |  |  |  |
| NPHP3 | RD |  | 7907 |  |  |  |  |
| NPHP4 | RD |  | 19104 |  |  |  |  |
| NPHS1 | RD |  | 7908 |  |  |  |  |
| NPHS2 | RD |  | 13394 |  |  |  |  |
| PDSS2 | RD |  | 23041 |  |  |  |  |
| PLA2R1 | RD |  | 9042 |  |  |  |  |
| PLCE1 | RD |  | 17175 |  |  |  |  |
| REG1A | RD |  | 9951 |  |  |  |  |
| REN | RD |  | 9958 |  |  |  |  |
| RPGRIP1L | RD |  | 29168 |  |  |  |  |
| SCARB2 | RD |  | 1665 |  |  |  |  |
| SDCCAG8 | RD |  | 10671 |  |  |  |  |
| SMARCAL1 | RD |  | 11102 |  |  |  |  |
| THBD | RD |  | 11784 |  |  |  |  |
| TMEM67 | RD |  | 28396 |  |  |  |  |
| TRPC6 | RD |  | 12338 |  |  |  |  |
| UMOD | RD |  | 12559 |  |  |  |  |
| XPNPEP3 | RD |  | 28052 |  |  |  |  |
| AI, Autoimmune; CT, Connective Tissue; HGNC, HUGO Gene Nomenclature Committee-approved gene identification number; HL, Hearing Loss; HSP, Hereditary Spastic Paraplegia; NS, Noonan Syndrome; Panel, targeted gene panel; Panel2, additional targeted gene panel including the corresponding gene; RD, Renal Disease. | | | | | | | |
